# Understanding smart home automation acceptance through users’ lifestyles and perceived difficulty of use: Evidence from South Korea

**DOI:** 10.1371/journal.pone.0352183

**Published:** 2026-06-26

**Authors:** Young-Gwan Kim, Do-Hyeon Ryu, Kwang-Jae Kim

**Affiliations:** 1 Department of Industrial and Management Engineering, Pohang University of Science and Technology, Pohang, Republic of Korea; 2 Department of Industrial and Management Engineering, Incheon National University, Incheon, Republic of Korea; Helwan University Faculty of Engineering, EGYPT

## Abstract

Smart home automation (SHA) is an advanced service that automates household tasks with minimal human intervention, enhancing efficiency, convenience, and personalization. SHA serves as the critical bridge that transforms home environments into intelligent spaces that respond adaptively to user needs. Despite these benefits, SHA penetration remains limited due to various challenges, making it essential to understand SHA acceptance. This study examines two user-centered constructs influencing SHA acceptance: users’ lifestyles (UL) and perceived difficulty of use (PDU). Through a systematic process, the dimensions of both constructs were defined and measurement items were developed. A research model extending the Technology Acceptance Model was proposed, in which UL and PDU influence the perceived usefulness (PU) and intention to use (IU) of SHA. The model was tested using data collected from 167 non-users of SHA in South Korea. Results reveal that UL characterized by energy-saving negatively affects IU, whereas UL characterized by lack of time for household tasks positively affects IU. Additionally, PDU from automation configuration negatively affects both PU and IU. Based on these findings, practical strategies to enhance SHA acceptance are proposed. This study contributes by extending the acceptance framework for SHA through conceptualizing UL and PDU and by revealing how these user-centered constructs shape PU and IU, thereby clarifying their role in SHA acceptance and informing managerial strategies that promote broader penetration.

## Introduction

The advancement of information and communication technologies (ICT) has introduced smart home automation (SHA) as a transformative service within smart homes, which are residential spaces where interconnected household devices, including lighting, security systems, and entertainment units, communicate and exchange data in real-time. SHA is a digital service that enables automated control of household appliances, minimizing manual operation by responding to predefined rules or real-time data [1]. For example, SHA can activate security systems, adjust thermostats, and turn off lights when users leave home. SHA improves household efficiency by automating device control based on user preferences, schedules, and environmental conditions, thereby reducing energy consumption and increasing convenience [2,3].

Despite these advantages, SHA penetration remains limited [4]. A recent survey revealed that 45% of homeowners rely on separate device apps, and 18% use direct controls to manage devices rather than utilizing SHA [5]. This limited penetration is closely related to user perceptions that SHA is unreliable or complex [6], highlighting that users are unsure whether SHA is truly necessary in their lives and tend to view its use as burdensome. These perceptions are captured by two user-centered concepts: users’ lifestyles (UL) and perceived difficulty of use (PDU). UL describes how individuals structure their daily activities and household routines in residential settings. Because SHA is designed to automate routine tasks that follow these daily patterns, a higher degree of fit between SHA functions and UL is likely to increase the necessity of SHA [7,8]. PDU captures users’ expectations about the effort required for SHA, including installation, rule design, troubleshooting, and ongoing adjustment. Because SHA requires users to make various active decisions and adjustments rather than receiving a predefined service, these tasks are easily anticipated as burdens. Such expected difficulties can form substantial anticipated barriers to acceptance, even when its advantages are already recognized [9].

Previous research has identified several factors that are relevant for explaining SHA acceptance, including privacy concerns, cost, and perceived technological complexity, but these factors have been examined mainly at the level of overall smart home technologies and environments rather than for SHA as a distinct service [10–12]. Because SHA is a user-driven service for managing daily routines, UL and PDU are particularly important for understanding whether and how users accept SHA. Nevertheless, UL and PDU remain underexplored in existing literature. Previous studies provide only indirect inferences about which lifestyles might benefit from SHA, and empirical validation of how UL influences SHA acceptance is limited. Similarly, the field lacks a systematic investigation of PDU and how PDU influences SHA acceptance.

This study investigates the impact of UL and PDU on SHA acceptance using data from non-users of SHA in South Korea. The research model conceptualizes acceptance through perceived usefulness (PU) and intention to use (IU), and examines how UL drives user acceptance of SHA and how PDU shapes acceptance among non-users. Beyond contributing empirical evidence on these relationships, the study aims to generate practical insights for SHA providers to develop effective strategies for increasing acceptance. The study proceeds as follows: The Literature review section reviews existing literature on SHA and establishes theoretical foundations for UL and PDU. The Research model development section outlines the research model development process for examining SHA acceptance. The Empirical analysis section presents empirical analysis results and discusses practical implications. The Concluding remarks section concludes with theoretical contributions and future research directions.

## Literature review

### Smart home automation and its acceptance

Smart home automation (SHA) refers to a service within the smart home that enables the automatic control and coordination of various household devices based on predefined user preferences, schedules, or environmental conditions. SHA operates through smart home assistants such as Amazon Alexa, Google Home, and Apple HomeKit, which allow users to configure automation rules that synchronize device operations according to predefined conditions or triggers. For example, a ‘Good Morning’ rule can activate at a preset time in the morning, automatically adjusting room temperature, brightening lights, and starting coffee brewing. SHA enhances daily life by reducing unnecessary energy consumption through automated control, improving convenience by minimizing manual device operation, and providing a personalized experience by adjusting settings to match user preferences. From a broader perspective, SHA functions as a foundational mechanism that underpins a wide range of smart home services, enabling capabilities such as energy management and safety monitoring while also promoting cross-device integration and fostering technological advancements across related industries [13–15].

Despite its potential advantages, SHA penetration remains limited. Understanding the factors that shape its acceptance is therefore an important research question. However, prior research has examined acceptance primarily at the level of overall smart home technologies and environments rather than for SHA as a distinct service. This leaves insufficient explanation of how SHA’s features connect to user-centered factors in shaping its acceptance. Within multiple theoretical frameworks such as the Technology Acceptance Model, UTAUT2, Value-based Adoption, and Innovation Diffusion Theory [12,16], critical determinants identified across studies include compatibility and perceived usefulness (PU) [4], perceived ease of use [17], and reliability of automation [18]. Primary motivations for acceptance include energy management, healthcare monitoring, and home security [19,20], while barriers comprise privacy, security risks, implementation costs, and operational complexity [10,11]. Trust [21,22], technology affinity [23], psychological factors [24], and demographic differences [17,25] have also been examined. However, two factors central to SHA acceptance remain insufficiently addressed: users’ lifestyles (UL), which can drive acceptance in distinct ways, and perceived difficulty of use (PDU), which can pose distinct barriers to acceptance at different stages of SHA use.

### Smart home automation acceptance: users’ lifestyles and perceived difficulty of use

This section examines UL and PDU as key factors influencing SHA acceptance.

#### Users’ lifestyles.

UL plays an important role in SHA because automation operates within users’ daily routines. The usefulness of SHA depends on how individuals organize their everyday activities, needs, and priorities. UL therefore shapes when automation becomes necessary, which tasks users expect automation to support, and how appropriate SHA appears in their daily living patterns. For instance, users who closely monitor household energy consumption may perceive greater value in SHA due to its ability to support energy-related control and scheduling [26]. However, even UL that appears favorable to SHA does not always lead to strong acceptance. This highlights the need not only to identify which UL is favorable, but also to understand whether such UL actually drives acceptance.

Prior research in technology acceptance has recognized UL as an important determinant of user behavior [27,28], and smart home studies similarly show that alignment between SHA features and UL enhances perceived value and supports acceptance [29–31]. Users tend to view SHA favorably when its functions fit or enhance their lifestyle [32,33]. More broadly, several studies have identified lifestyle compatibility as a key property and source of value in the smart home contexts [23,34]. Building on this literature, this study identifies three dimensions of UL particularly relevant to SHA acceptance in everyday home contexts: structured routines, energy-saving, and lack of time for household tasks.

Energy-saving lifestyles, characterized by efforts to conserve energy and concerns about excessive energy usage, drive SHA acceptance. Users who prioritize energy efficiency tend to show stronger interest in home automation technologies [8,35,36]. Energy-conscious users may find SHA valuable for real-time energy monitoring, automated power control for idle devices, and context-aware recommendations aligned with their conservation habits.

Structured routines, such as regular sleeping and waking, consistent home entry and exit times, and consistent daily tasks, create favorable conditions for SHA acceptance. Research demonstrates that automated home systems work most effectively when aligned with these structured household behavioral patterns [37–39]. When users follow predictable schedules, SHA can provide timely support. For example, it may adjust lighting based on sleep and wake cycles or activate security systems based on regular departure and arrival times.

Lack of time for household tasks serves as a practical driver for SHA acceptance, particularly among individuals with frequent external activities, excessive schedules, or inadequate allocation of time for household tasks. For these users, SHA provides meaningful value by enabling remote and autonomous control of appliances. It supports efficient task management at early-morning or late-evening hours, when users have limited daytime availability, and automates device operations when users are occupied elsewhere [40,41].

#### Perceived difficulty of use.

PDU is a key barrier to SHA acceptance because SHA operates as a user-driven service. Instead of passively receiving a predefined service, users must be involved throughout an extended process that includes selecting products, designing and revising automation rules, configuring systems, troubleshooting errors, and performing ongoing maintenance. Each stage can impose cognitive and technical burdens [42], meaning that users evaluate not only the benefits of SHA but also the effort required to obtain them [10].

PDU refers to the degree to which users believe that accepting a system requires considerable effort and involves complex processes [43]. Technology-acceptance research consistently identifies PDU as a major barrier, encompassing technical complexity, configuration difficulty, and the cognitive effort needed for system management [44]. Empirical studies show that higher PDU tends to reduce both PU and intention to use (IU) of smart home services [10,45]. For instance, configuration complexity and inadequate user support often lead to user withdrawal during early acceptance stages [46], while maintenance-related frustrations such as system malfunctions and poor interoperability hinder sustained acceptance [47,48]. These findings indicate that PDU significantly affects SHA acceptance.

Building on this literature, this study identifies three dimensions of PDU, organized by the stages at which barriers typically emerge during SHA use. The first dimension, Product and Infrastructure Setup, involves device selection and hardware preparation, which is often complicated by interoperability issues and insufficient infrastructure guidance [46,49]. The second dimension, Automation Configuration, concerns the design and implementation of automation rules based on user needs; this stage is frequently challenging due to the abstract nature of trigger-action logic and limited support for error handling [50]. The third dimension, Management and Maintenance, relates to ongoing system operation, which imposes a continuous burden from software errors, device instability, and inadequate customer support [47].

### Research gap

The Users’ lifestyles and Perceived difficulty of use sections reviewed prior work on UL and PDU as factors relevant to SHA acceptance. While these studies provide valuable conceptual grounding, important gaps remain in how UL and PDU have been examined in the SHA context. SHA differs from conventional smart home devices in that it requires active user involvement throughout the entire use process, from initial setup to ongoing management, and prior research has not adequately accommodated these distinct characteristics when investigating the role of UL and PDU. Table 1 summarizes prior empirical work on smart home and SHA acceptance, showing that UL and PDU have not been jointly examined in a structured, comparative manner.

**Table 1:** Summary of prior studies on smart home and SHA acceptance and their coverage of UL and PDU.

Study	Sample	Scope	Methods	Examined variables	UL / PDU coverage
Hubert et al. [4]	n = 409, Germany	Smart home	Structural equation modeling integrating Technology Acceptance Model, Innovation Diffusion Theory, and Perceived Risk Theory	Perceived usefulness, perceived ease of use, compatibility, trialability, result demonstrability, visibility, perceived security/performance/time risks	UL: None (Compatibility addresses value consistency, not lifestyle.) PDU: Partial (Reduced to perceived ease of use, with perceived risks treated separately; the multidimensional procedural challenges of SHA are not captured.)
Shin et al. [17]	n = 310 (survey 1) and n = 2,113 (survey 2), South Korea	Smart home	Structural equation modeling with extended Technology Acceptance Model, complemented by multivariate probit analysis	Compatibility, perceived ease of use, perceived usefulness, privacy concerns; demographic and household ICT possession factors	UL: None (Compatibility addresses value consistency, not lifestyle.) PDU: Partial (Reduced to perceived ease of use, with privacy concerns treated separately; the multidimensional procedural challenges of SHA are not captured.)
Schomakers et al. [18]	n = 137, Germany	SHA	Adaptive choice-based conjoint analysis with six smart home product attributes	Application area, data type, data storage location, automation reliability, awareness source, automation level	UL: None (Lifestyle not addressed.) PDU: None (Automation reliability is a trust attribute, not user-perceived difficulty.)
Valencia-Arias et al. [12]	12 studies	Smart home	Systematic literature review	Perceived reliability, perceived usefulness, perceived ease of use, perceived behavioral control, perceived enjoyment	UL: None (Lifestyle not addressed.) PDU: Partial (Reduced to perceived ease of use; the multidimensional procedural challenges of SHA are not captured.)
Chang & Nam [25]	n = 400, South Korea	Smart home	Hierarchical regression analysis	Service preferences across convenience, safety, energy, and healthcare domains; demographic and residential factors	UL: None (Lifestyle not addressed.) PDU: None (Service preferences address consumer choice, not user-perceived difficulty.)
Nascimento et al. [16]	24 studies	Smart home	Meta-analysis based on UTAUT2	Performance expectancy, effort expectancy, social influence, facilitating conditions, hedonic motivation, price value, attitude, perceived risk; gender and publication year as moderators	UL: None (Lifestyle not addressed.) PDU: Partial (Reduced to effort expectancy; the multidimensional procedural challenges of SHA are not captured.)
Wang et al. [45]	n = 387, China	Smart home	Structural equation modeling integrating Technology Acceptance Model and Innovation Diffusion Theory	Compatibility, trialability, observability, perceived ease of use, perceived usefulness, intergenerational technical support, perceived cost, self-reported health	UL: None (Compatibility and self-reported health address other constructs, not lifestyle.) PDU: Partial (Reduced to perceived ease of use, with perceived cost treated separately; the multidimensional procedural challenges of SHA are not captured.)
Ferreira et al. [26]	n = 255, multinational	Smart home	Structural equation modeling based on UTAUT2	Performance expectancy, effort expectancy, social influence, facilitating conditions, hedonic motivation, price value, habit, environmental awareness	UL: Partial (Environmental awareness and habit added as UTAUT2 extensions, not a structured set of UL dimensions for SHA.) PDU: Partial (Reduced to effort expectancy; the multidimensional procedural challenges of SHA are not captured.)

As Table 1 indicates, the literature has predominantly examined acceptance of smart home technologies in general. Across these studies, UL has either been omitted entirely or captured only through narrow proxies such as habit or environmental awareness, rather than as a structured representation of lifestyle patterns relevant to SHA. PDU has most often been reduced to perceived ease of use, with related issues such as perceived risks and perceived cost examined as separate variables rather than integrated as components of a multidimensional construct reflecting the procedural challenges of SHA.

Two gaps stand out. First, regarding UL, existing studies have generally discussed lifestyle in broad terms without organizing it into distinct patterns relevant to SHA, and empirical investigation into how individual UL dimensions relate to PU and IU has been largely absent. This gap makes it difficult to identify which types of users are more likely to accept SHA, an understanding essential for promoting acceptance across different user groups. Second, regarding PDU, prior research has frequently conceptualized difficulty through the broad notion of perceived ease of use, treating it as a single, undifferentiated construct. This approach fails to reflect the procedural challenges involved in SHA, which emerge across multiple stages from initial setup through ongoing management, and obscures the challenges that arise at different stages of SHA use. A structured understanding of PDU is necessary to identify concrete intervention points and to facilitate broader SHA acceptance.

To address these gaps, the present study examines UL as a set of conditions that may favor SHA acceptance and empirically investigates their associations with PU and IU. In parallel, it defines PDU as a multidimensional construct that captures not only technical obstacles but also the cognitive burdens that users associate with implementing and operating SHA, and empirically examines the separate effects of the three PDU dimensions on PU and IU. By jointly modeling UL and PDU, the study provides an empirical account of how these two factors shape SHA acceptance in a structured, comparative manner that prior research has acknowledged in conceptual terms but has not yet examined empirically.

## Research model development

**Fig 1:**
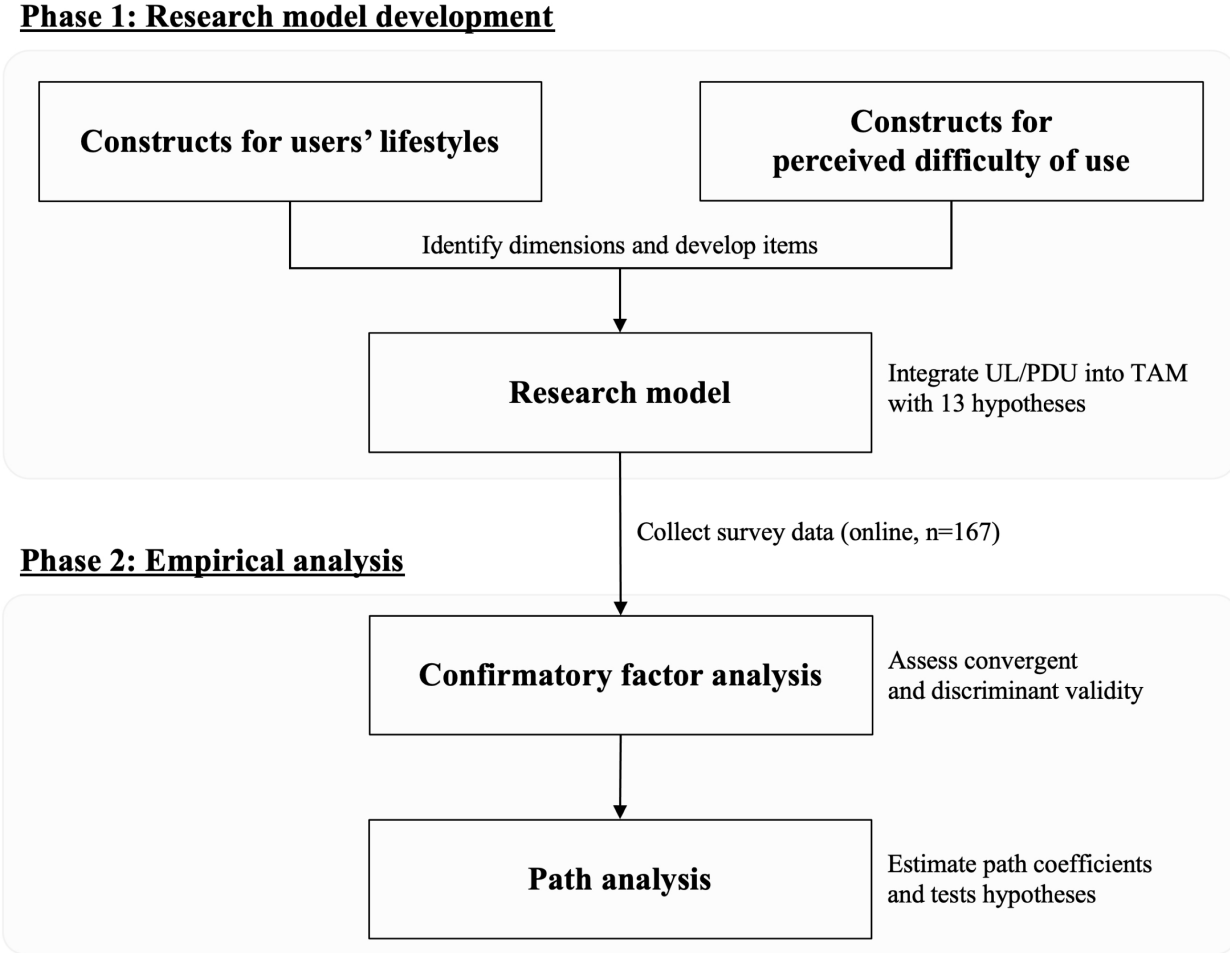
Overall research workflow.

As illustrated in Fig 1, the overall research workflow consists of two major phases corresponding to the Research model development and Empirical analysis sections of this study: research model development and empirical analysis. The Research model development section presents the research model development process, while the Empirical analysis section presents the empirical analysis of the proposed model through confirmatory factor analysis (CFA) and path analysis. CFA is conducted to assess convergent and discriminant validity, while path analysis is performed to examine the hypothesized relationships among users’ lifestyles (UL), perceived difficulty of use (PDU), perceived usefulness (PU), and intention to use (IU).

The research model development follows two steps to examine the influence of UL and PDU on SHA acceptance. First, the dimensions and measurement items of UL and PDU are developed. Second, a research model is established by positioning UL and PDU as central constructs and formulating hypotheses about their effects on SHA acceptance. This model serves as an analytical framework for testing relationships among these constructs.

### Constructs for users’ lifestyles

The development of UL constructs followed a three-step process: literature-based dimension identification (the Users’ lifestyles section), item operationalization grounded in prior research, and expert content validation. The three dimensions identified in the Users’ lifestyles section are energy-saving (ES), structured routines (SR), and lack of time for household tasks (LT).

ES contains two items: efforts to conserve energy (ES1) and concerns about excessive energy usage (ES2). Energy-saving lifestyle manifests in active behaviors such as powering off devices when not in use, using lower-energy settings, replacing appliances with more efficient alternatives, and shifting use to off-peak periods, which together describe the practical actions through which households reduce consumption [51]. Beyond these actions, households also differ in how concerned they are about using too much energy, whether environmentally or financially, and this concern represents a separate facet of energy-saving lifestyle distinct from active behavior [52]. Together, the two items capture the behavioral and attitudinal facets through which energy-saving lifestyle is expressed in daily life.

SR contains three items: regular sleeping/waking (SR1), regular entry/exit (SR2), and consistent daily tasks (SR3). The regularity of sleeping and waking schedules establishes predictable morning and evening transitions, which form the most stable rhythm in daily routines [53,54]. The regularity of home entry and exit captures the predictability of when household members are present or absent, providing direct signals for security activation, climate adjustment, and arrival-based automation rules. Consistent daily tasks such as meals, cleaning, and exercise form recurring activity patterns that reflect the stability of daily life. Together, these items capture the temporal regularity through which households’ daily lives become aligned, or misaligned, with the predictability that SHA requires to deliver value.

LT contains three items: frequent external activities (LT1), excessive schedules (LT2), and inadequate allocation of household task time (LT3). Frequent external activities such as work, social commitments, and errands reduce the time households can spend at home managing routine tasks, which raises the value of remote and autonomous appliance control. Excessive schedules reflect chronic time pressure, an ongoing experience of time shortage and feeling rushed that pervades modern household life [55]. Inadequate allocation of household task time captures the residual problem when work and external commitments crowd out the time available for domestic work, in which case households become more receptive to SHA as a practical solution.

To assess content validity, the operationalized items were reviewed by two experts with research and practice backgrounds in smart home services and user experience. Their review focused on whether each item adequately represented the underlying lifestyle pattern in a smart home context, item wording, and conceptual overlap. Items were revised in wording before the final scales were deployed. The resulting operational definitions and items are presented in Table 2.

**Table 2:** Identified dimensions and items for ULs and PDUs.

Type	Dimension (Code)	Description	Item (Code)
Users’ lifestyles (UL)	Energy-saving (ES)	The lifestyle that prioritizes energy conservation and reflects a conscious effort to reduce energy consumption	Efforts to conserve energy (ES1)
			Concerns about excessive energy usage (ES2)
	Structured routines (SR)	The lifestyle that follows consistent and predictable daily patterns, emphasizing regularity in activities	Regular sleeping/waking (SR1)
			Regular entry/exit (SR2)
			Consistent daily tasks (SR3)
	Lack of time for household tasks (LT)	The lifestyle that reflects limited availability for managing household	Frequent external activities (LT1)
			Excessive schedules (LT2)
			Inadequate allocation of household task time (LT3)
Perceived difficulty of use (PDU)	Product and Infrastructure Setup (PS)	Difficulties in setting up and understanding infrastructure	Selecting products and services (PS1)
			Insufficient infrastructure information (PS2)
			Lack of additional resources (PS3)
			Building infrastructure (PS4)
	Automation Configuration (AC)	Difficulties in designing and configuring automation routines	Designing automation (AC1)
			Configuring automation (AC2)
	Management and Maintenance (MM)	Difficulties in managing and maintaining smart home automation	Emotional barrier (MM1)
			Problem-solving (MM2)
			Continuous management (MM3)

### Constructs for perceived difficulty of use

The development of PDU constructs followed a four-step process: literature-based dimension identification (the Perceived difficulty of use section), a supplementary qualitative survey of heavy SHA users to examine the dimensions and gather background for item development, item operationalization grounded in prior research, and expert content validation. The supplementary qualitative survey was conducted for PDU but not for UL because the two constructs differ in how their dimensions are grounded. UL dimensions describe general lifestyle characteristics that are well-established in broader research on household behavior, energy use, and time use, and they exist in households regardless of SHA experience. Expert content validation alone can ensure the items capture these characteristics. PDU dimensions, by contrast, describe difficulties that arise only during actual SHA use, and examining whether the literature-derived PDU dimensions correspond to the difficulties Korean SHA users actually encounter required input from respondents with direct SHA usage experience. This step concerns the dimensional structure rather than measurement of perceived difficulty of use itself. The three dimensions identified in the Perceived difficulty of use section are Product and Infrastructure Setup (PS), Automation Configuration (AC), and Management and Maintenance (MM).

A supplementary qualitative survey was conducted in March 2024 with 13 heavy SHA users recruited from an online smart home community, with informed consent obtained through the same procedure as the main survey described in the Empirical analysis section. This survey served two purposes: to qualitatively examine whether the three dimensions identified in the literature review corresponded to the difficulties SHA users encounter in South Korea, and to provide concrete background that informed the wording and scope of the items developed in the next step. Participants’ responses covered all three dimensions identified in the literature review and provided concrete examples that informed the item operationalization presented in the following paragraphs. The three dimensions were established a priori from the literature review (the Smart home automation acceptance: users’ lifestyles and perceived difficulty of use section); the qualitative survey was therefore not used as a source for the construct structure of PDU but only to confirm its applicability to South Korea and to inform the development of survey items.

Heavy users were appropriate for this role because qualitatively examining whether the three dimensions arise across all stages of SHA use, and gathering examples of how they manifest in practice, requires respondents with direct experience of every phase from product selection to long-term maintenance. While these heavy users may possess higher technical self-efficacy than the general non-user population, the difficulties captured by the PDU dimensions are not unique to enthusiasts and arise across users of varying experience levels. Within each dimension, the form of difficulty ranges from lower-level obstacles for less familiar users to more technical challenges for experienced users. Because SHA operates as a user-driven service requiring active user involvement throughout the entire use process, prior research has documented that product selection complexity, automation configuration burden, and ongoing maintenance demands are encountered by general and technically inexperienced households as well, with differences across user types lying in the severity and resolution speed of these difficulties rather than in their occurrence [8,42,46,56].

PS contains four items: selecting products and services (PS1), insufficient infrastructure information (PS2), lack of additional resources (PS3), and building infrastructure (PS4). The smart home market offers a wide variety of products with limited cross-compatibility and standardization, and households face difficulty in choosing suitable products without guidance on interoperability and reliability [57]. Households also lack the technical knowledge to assess infrastructure requirements such as network protocols (Wi-Fi, Zigbee, Z-Wave) and power needs, leading to incomplete or failed setups. Households rely on a mix of supplementary resources such as manuals, technical guides, and customer support during setup, but the resulting learning curve still makes setup a slow and effortful process that requires sustained time and trial-and-error effort [58]. Finally, configuring hubs, sensors, and wiring during physical installation requires technical proficiency that many households do not possess, often necessitating professional assistance.

AC contains two items: designing automation (AC1) and configuring automation (AC2). Designing effective automation rules requires households to translate their needs into structured trigger-action logic, and many households struggle with the abstract nature of rule semantics, anticipating exceptional cases and predicting how multiple automations interact [59]. Configuring automation involves additional technical complexity such as navigating system settings, integrating devices across platforms, and debugging unexpected behavior [57]. A substantial proportion of user support requests in active SHA use concerns automation configuration, indicating that this stage remains technically and cognitively demanding even for experienced users.

MM contains three items: emotional barrier (MM1), problem-solving (MM2), and continuous management (MM3). Households experience emotional burdens such as frustration, regret, and anger when SHA performance falls short of expectations or when malfunctions arise, and these negative emotional responses undermine sustained use of the technology [60]. Diagnosing and resolving system failures is technically demanding, and household members who did not initiate the technology must nonetheless take on troubleshooting tasks such as diagnosing, performing fixes, or delegating to external support, as part of unacknowledged digital housekeeping work [61]. Beyond reactive problem-solving, the continuous effort required to maintain SHA, such as software updates, hardware replacements, and adjustments to accommodate lifestyle changes, imposes an ongoing burden on households with limited time or technical skills.

To assess content validity, the items were reviewed by the same two experts who validated the UL items. Their review focused on whether each item adequately represented the underlying procedural difficulty in a smart home context, item wording, and conceptual overlap. Items were revised in wording before the final scales were deployed. The resulting operational definitions and items are presented in Table 2.

### Research model

**Fig 2:**
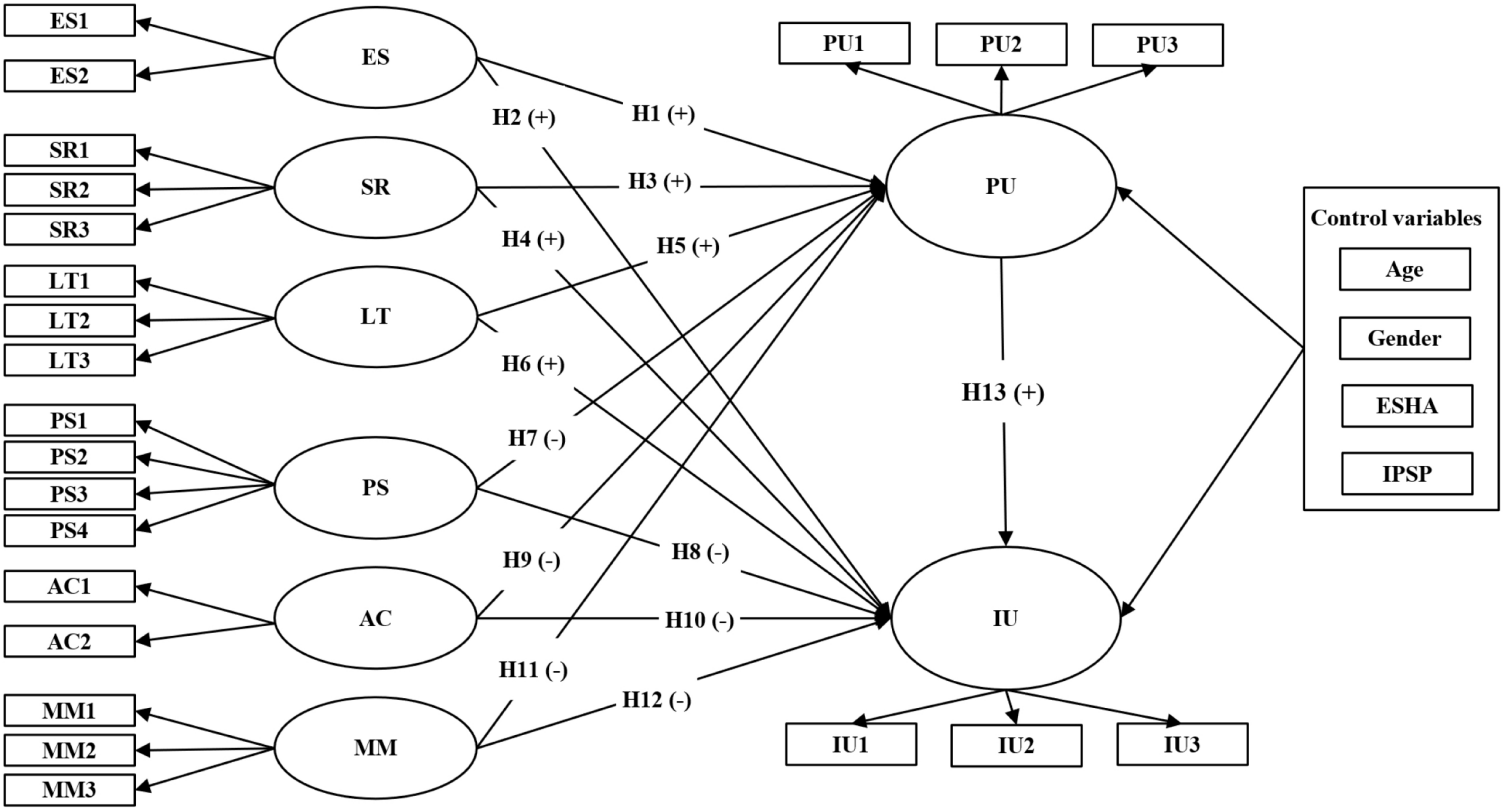
Proposed research model.

This study develops a research model (Fig 2) to examine the impact of the proposed UL and PDU constructs on SHA acceptance. The model is based on the Technology Acceptance Model (TAM) [62], incorporating hypotheses regarding the effects of UL and PDU on the PU of SHA and the IU toward SHA. The original TAM specifies PU and perceived ease of use (PEOU) as core determinants of acceptance but does not specify which external variables drive these perceptions. Earlier extensions of acceptance models in smart home research, as reviewed in the Smart home automation and its acceptance section and Table 1, have not addressed how UL drives SHA acceptance or how PDU of SHA extends beyond general ease of use.

The present study extends TAM in two ways. First, it introduces UL and PDU as new external variables tailored to SHA: UL captures how users’ everyday routines and priorities shape acceptance and drives both PU and IU, while PDU captures procedural difficulties from product selection through ongoing management and complements PEOU by representing difficulty as multidimensional rather than as a single ease of use measure. Second, each dimension of UL and PDU is modeled as a separate external variable, enabling the effect of each dimension on PU and IU to be tested individually rather than as part of a combined construct. This structured, multidimensional treatment of UL and PDU constitutes the model’s central theoretical advancement.

PU is defined as the degree to which an individual believes that using SHA will enhance energy efficiency, provide convenience, and offer a personalized experience. IU represents the likelihood that an individual will intend to use SHA. As the dependent variable in this study, IU captures the planned or intended future use of SHA rather than actual use. Since actual use requires longitudinal observations, IU is widely employed as a key predictor in new technology acceptance research [63,64]. IU is assessed using items related to consideration of use, willingness to use, and intention to recommend SHA [25].

Because the present study targets non-users of SHA, actual use behavior cannot be observed by design, and post-acceptance phenomena such as continued usage, abandonment, and maintenance burden lie beyond its scope. This focus is appropriate given the current state of SHA, where adoption remains limited [65] and understanding what shapes non-users’ acceptance is essential to advancing it. While actual use research offers important insights into such phenomena, several of these concerns can also be examined at the pre-acceptance stage; the present study addresses them by examining how users’ perceived procedural difficulties across stages from product selection to ongoing system management shape their intention to use SHA. PU is positioned as a mediating construct that links UL and PDU to IU. However, UL and PDU may also have direct effects on IU, as they can independently influence users’ willingness to use SHA. By incorporating both direct and mediated pathways, the model provides a more comprehensive understanding of the relationships among UL, PDU, PU, and IU.

The research model adopts a first-order factor structure in which each UL dimension (ES, SR, LT) and each PDU dimension (PS, AC, MM) is modeled as an independent external variable of TAM. This structure is appropriate for the present study for two reasons. First, the central research question concerns which dimensions of UL and PDU shape PU and IU, and with what direction and magnitude. A first-order structure directly estimates the separate path from each dimension to the outcomes, whereas a second-order structure would collapse the dimensions into a single composite path and obscure the effects of each dimension that the present study is designed to identify.

Second, the dimensions of UL and PDU each carry a distinct influence on PU and IU, requiring separate examination to identify the contribution of each dimension. For UL, structured routines, energy-saving, and lack of time for household tasks reflect different aspects of daily life that shape SHA acceptance to different degrees and through different channels. For PDU, product and infrastructure setup, automation configuration, and management and maintenance involve cognitively different difficulties at different stages of SHA, and the strength of their negative effect on acceptance is likely to differ across stages. Aggregating the dimensions into a higher-order composite would conflate these dimension-specific influences and obscure which dimension carries the strongest effect, whereas a first-order structure preserves them, consistent with prior research that examines sub-dimensions through individual paths to outcomes when they exert qualitatively different effects [66,67].

The following hypotheses are proposed to examine the relationships among UL, PDU, PU, and IU in the context of SHA acceptance:

<UL>
H1: ES positively influences PU. H2: ES positively influences IU.
H3: SR positively influences PU. H4: SR positively influences IU.
H5: LT positively influences PU. H6: LT positively influences IU.
<PDU>
H7: PS negatively influences PU. H8: PS negatively influences IU.
H9: AC negatively influences PU. H10: AC negatively influences IU.
H11: MM negatively influences PU. H12: MM negatively influences IU.
<PU>
H13: PU positively influences IU.

The model incorporates personal characteristics as control variables. Age and gender are fundamental demographic variables commonly used to differentiate users in technology acceptance research, as they influence perceptions and behaviors toward new technologies [68,69]. Prior experience with SHA (ESHA) also serves as a control variable because familiarity with automation technologies may enhance perceived usefulness, consequently leading to higher IU [70,71]. Additionally, interests in products and services for personalization (IPSP) serves as a control variable since users with strong personalization preferences may recognize greater practical value in SHA [72].

## Empirical analysis

This section presents the empirical analysis conducted to validate the research model. Data were collected through an online survey distributed across various platforms. The first page of the survey presented an information sheet describing the study purpose, the voluntary nature of participation, the anonymous handling of responses, the right to withdraw at any time, and the use of responses solely for academic research. Respondents could proceed only after providing informed consent through a mandatory online checkbox. Only age and gender were collected as demographic information, and no personally identifying information was recorded. The data collection took place in July 2024, initially gathering 180 responses. After excluding incomplete or inappropriate responses, 167 valid responses were used for analysis. Table 3 presents the demographic characteristics of the final respondents, offering a comprehensive overview of the study sample. The survey employed a 7-point Likert scale, allowing participants to express nuanced perceptions and attitudes toward smart home automation (SHA).

**Table 3:** Demographic information of the final respondents.

Type	Age					
	10s	20s	30s	40s	50s	60s
Male	4	28	19	16	11	5
Female	2	45	14	9	12	2
Total (%)	6 (3.6)	73 (43.7)	33 (19.8)	25 (15.0)	23 (13.8)	7 (4.2)

To ensure alignment with the study’s objectives, respondents were limited to non-users of SHA. While participants may or may not have experience with smart home environments, restricting the sample to non-users of SHA was necessary to accurately analyze the influence of users’ lifestyles (UL) and perceived difficulty of use (PDU) on SHA acceptance. To provide clarity, the online survey included detailed explanations of SHA and the proposed UL and PDU constructs before participants answered related items. These descriptions ensured that respondents had a clear understanding of SHA functionalities and the specific dimensions of UL and PDU, enhancing the reliability of their responses. Each participant rated their perceptions and behaviors concerning these constructs.

### Confirmatory factor analysis

The research model was evaluated using Confirmatory factor analysis (CFA) to assess the validity and reliability of the measurement constructs. The analysis focused on ensuring that the items accurately measured their respective latent constructs and that the dimensions were distinct from one another. In particular, construct validity, including convergent and discriminant validity, was examined to confirm the robustness of the measurement model.

Convergent validity was assessed using four key criteria: factor loadings, squared multiple correlations (SMC), average variance extracted (AVE), and composite reliability (CR). Factor loadings indicate the strength of the relationship between each observed item and its corresponding construct, with a loading of 0.7 or higher considered desirable [73]. SMC evaluates how well each item explains the variance in its construct, with values above 0.5 indicating strong explanatory power [74]. AVE measures the proportion of variance in the items captured by the construct relative to measurement error, with values of 0.5 or greater supporting convergent validity [75]. CR examines internal consistency, with values above 0.7 indicating that the items reliably measure their construct [76]. The results, summarized in Table 4, show that all constructs satisfied the recommended thresholds, confirming that the measurement items effectively represent their respective constructs.

**Table 4:** Results of convergent validity assessment.

Type	Dimension	Item	Factor loading	SMC	AVE	CR
UL	ES	ES1	0.77	0.60	0.54	0.70
		ES2	0.70	0.49		
	SR	SR1	0.69	0.47	0.54	0.78
		SR2	0.79	0.62		
		SR3	0.72	0.52		
	LT	LT1	0.72	0.52	0.50	0.75
		LT2	0.76	0.58		
		LT3	0.64	0.41		
PDU	PS	PS1	0.74	0.55	0.59	0.85
		PS2	0.78	0.62		
		PS3	0.79	0.63		
		PS4	0.76	0.57		
	AC	AC1	0.83	0.69	0.67	0.80
		AC2	0.81	0.65		
	MM	MM1	0.66	0.43	0.54	0.78
		MM2	0.79	0.62		
		MM3	0.75	0.57		

Discriminant validity was evaluated by comparing the square root of each construct’s AVE with the intercorrelations among constructs. Discriminant validity is established when the square root of a construct’s AVE exceeds its correlations with other constructs, indicating that the constructs are distinct [75]. The results, presented in Table 5, confirm that the model’s constructs are independent, supporting the robustness of the measurement model.

**Table 5:** Result of discriminant validity assessment.

	ES	SR	LT	PS	AC	MM
ES	0.74					
SR	0.11	0.74				
LT	0.07	0.41	0.72			
PS	0.08	0.18	0.24	0.77		
AC	0.10	0.27	0.40	0.76	0.83	
MM	0.32	0.01	0.29	0.67	0.65	0.74

To further support the structural choice on empirical grounds, the first-order model was compared with a second-order model and a hybrid configuration in which one construct was modeled as a second-order factor. The first-order model demonstrated the best fit, with normed chi-square (1.60), goodness of fit index (GFI; 0.86), adjusted goodness of fit index (AGFI; 0.80), comparative fit index (CFI; 0.92), and root mean square error of approximation (RMSEA; 0.06) all meeting commonly accepted thresholds. These fit results empirically support the structural choice articulated in the Research model section.

Overall, these results confirm that the measurement model is statistically robust, providing a reliable foundation for the subsequent path analysis.

### Path analysis

**Fig 3:**
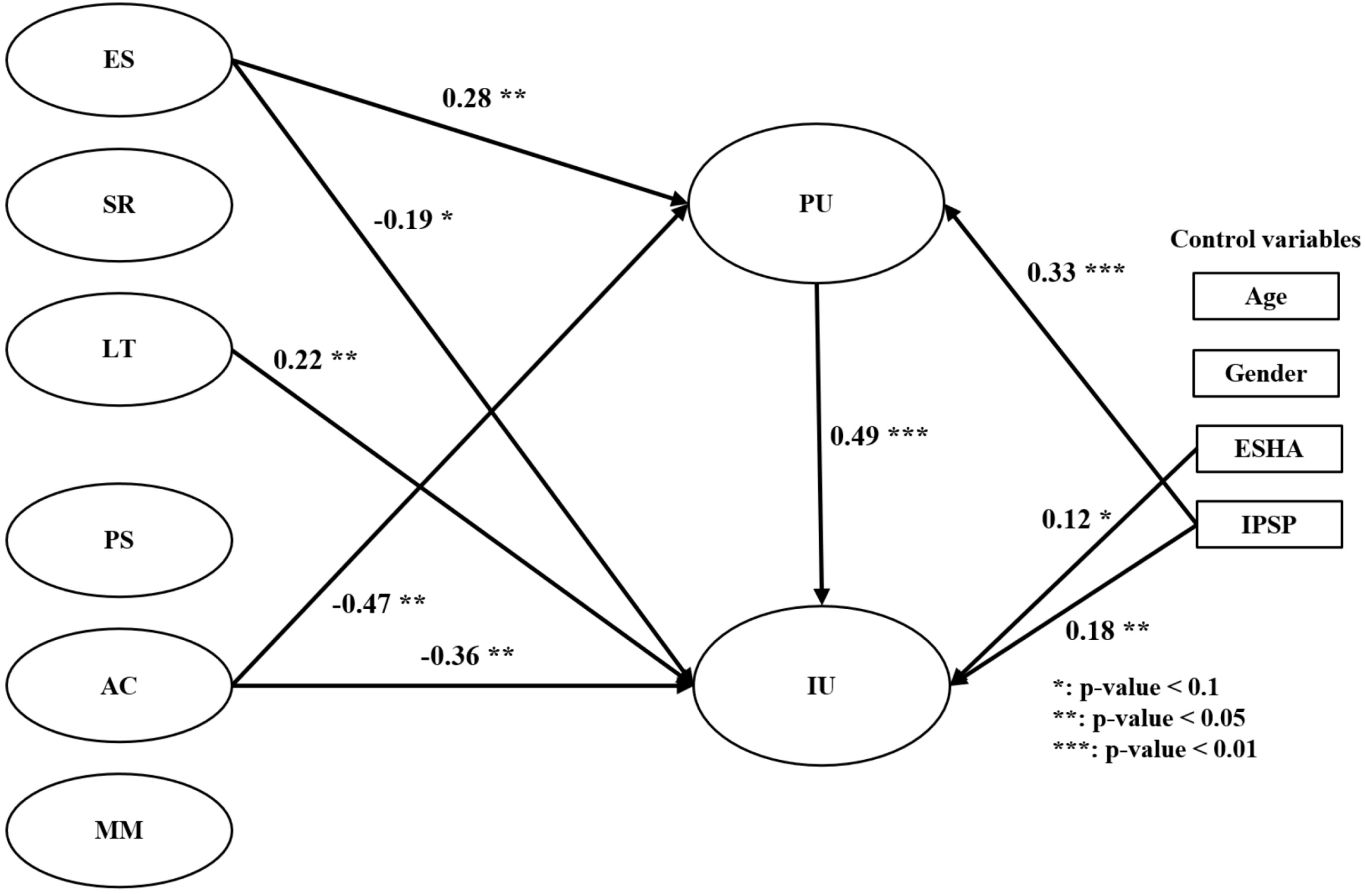
Significant paths in the model.

Path analysis was conducted using structural equation modeling (SEM) to examine the relationships between UL, PDU, PU, and IU while accounting for individual differences through control variables. SEM was chosen for its ability to simultaneously estimate multiple relationships among latent constructs and observed variables, ensuring a robust analysis of the proposed model. The results, summarized in Table 6 and Fig 3, highlight significant relationships between the constructs. Given the exploratory nature of this study, p-values of <0.001, < 0.05, and <0.1 were considered significant, with corresponding significant paths bolded in Table 6.

**Table 6:** All path coefficients in the model.

Dimension and control variable	Path to PU	Path to IU	
	Coefficient (p-value)	Coefficient (p-value)	
UL-ES	**0.28 (0.016)**	**−0.19 (0.062)**	
UL-SR	0.05 (0.642)	0.11 (0.260)	
UL-LT	0.18 (0.153)	**0.22 (0.040)**	
PDU-PS	0.24 (0.220)	0.03 (0.848)	
PDU-AC	**−0.47 (0.016)**	**−0.36 (0.041)**	
PDU-MM	0.08 (0.651)	0.14 (0.366)	
PU	–	**0.49 (<0.001)**	
Age	0.05 (0.496)	−0.04 (0.510)	
Sex	0.10 (0.193)	0.00 (0.993)	
ESHA	−0.03 (0.714)	**0.12 (0.059)**	
IPSP	**0.33 (<0.001)**	**0.18 (0.035)**	

Within the UL dimensions, ES positively influences PU (0.28) while showing a negative direct effect on IU (−0.19). LT also demonstrates a positive effect on IU (0.22). In contrast, SR does not show significant effects on either PU or IU. Among the PDU dimensions, AC significantly reduces both PU (−0.47) and IU (−0.36), whereas PS and MM exhibit no significant effects on either PU or IU. PU functions as a mediating variable in the model, exerting a strong positive effect on IU (0.49). Regarding control variables, IPSP shows positive effects on both PU (0.33) and IU (0.18). ESHA also has a positive effect on IU (0.12), although its effect on PU is not significant. Detailed interpretations of these results are provided in the Discussion section, particularly in Key findings section.

## Discussion

This study provides key insights into the factors influencing the SHA acceptance, focusing on the roles of users’ lifestyles (UL) and perceived difficulty of use (PDU). The findings highlight significant relationships between these factors and perceived usefulness (PU) and intention to use (IU). The Key findings section presents the key empirical findings by analyzing how each dimension of UL and PDU, as well as the control variables, influences PU and IU. The Managerial implications section builds on these results to propose four managerial implications from the perspective of smart home automation (SHA) providers, aimed at promoting acceptance.

### Key findings

#### Relationship between users’ lifestyles and intention to use.

ES exhibited a positive effect on PU but a negative direct effect on IU. ES users recognized SHA as a potentially useful tool. They generally strive to reduce unnecessary energy expenses in their daily lives, and this is usually linked to attention to usefulness of SHA. However, ES is simultaneously associated with a lower IU because ES users tend to apply stricter economic acceptance criteria. They may reject SHA if the expected savings are small, uncertain, or insufficient relative to the installation, maintenance, or learning costs over the long term. In markets such as South Korea, where household electricity costs are relatively low [77], the financial benefits that SHA can realistically deliver are modest, which reduces the practical motivation for acceptance. Prior research on energy-efficient technologies shows that IU strongly depends on the expected payback period, energy cost savings, and perceived cost-benefit trade-offs [78–80]. They may perceive SHA as useful in principle while simultaneously questioning whether the expected total reduction in electricity bills is large enough to justify the associated burdens [81–84]. As a result, acceptance of SHA increases only when the benefits are not merely visible but also substantial and sustained over time [85–88]. Beyond cost-benefit calculations, ES users may also be concerned about the energy that SHA itself consumes. SHA keeps hubs, sensors, and connected devices powered continuously, and prior research identifies this standby consumption, along with the possibility that automated convenience weakens active conservation behavior, as a recognized risk of SHA acceptance [19,89]. Given that ES is defined by attention to wasted energy, such users may weigh not only what SHA can save but also what SHA itself draws. This additional concern gives ES users a further reason to discount IU even when they recognize SHA as useful. Consequently, ES can contribute to higher IU only through the indirect path via PU (ES → PU → IU).

SR did not show a significant effect on either PU or IU. The results indicate that this is linked to the way daily routines actually unfold and to users’ shifting expectations for SHA. Domestic routines, even when they appear regular, are negotiated, contingent, and frequently disrupted, so they do not translate neatly into simple time-based rules or fixed schedules that can be automated in a stable way [90]. This implies that even users with highly regular lifestyles cannot easily translate their routines into stable, fully predictable automation rules, which limits the extent to which SR alone can raise PU of SHA. At the same time, users expect SHA to provide flexible autonomy, remote adjustability, and noticeable improvements in performance, and they are skeptical of services that only replicate existing routines without delivering clear added value [91]. This aligns with the present finding that SR does not automatically translate into higher PU or IU. Recent studies emphasize the core value of SHA as providing context-aware and personalized services that continuously learn and adapt to users’ habits, rather than merely executing fixed routines [92,93]. From this perspective, SHA is likely to be perceived as distinctly useful only when it offers adaptive, context-aware functionality beyond what simple routine-based automation can provide. Consequently, SR no longer functions as a strong standalone driver of PU or IU for SHA in the current market and technological landscape.

LT has a positive effect on IU, but no significant effect on PU. This indicates that IU is driven mainly by problem awareness (“I do not have enough time for housework”) rather than by a belief that SHA will be useful. Time saving and reduced effort are frequently emphasized in SHA promotions, especially for dual-income or busy households, which makes SHA appear attractive as a potential solution for people who feel chronically short of time [42,94,95]. However, these users often lack concrete evidence that SHA will reliably reduce their workload. The same studies also report substantial concerns regarding complexity, learning effort, and limited or situation-dependent benefits. LT therefore generates a problem-driven IU based on hope and general expectations, while evaluative beliefs about usefulness remain similar across users with high and low LT. This also highlights a clear opportunity. Users with high LT already have strong incentive to use SHA, but their PU has not yet caught up with their intention. If these users come to perceive SHA as genuinely useful, an additional positive pathway (LT → PU → IU) would be established. Clearly demonstrating how SHA reduces effort in everyday life is therefore essential.

#### Relationship between perceived difficulty of use and intention to use.

PS shows no significant effect on PU or IU. This indicates that difficulties from product and infrastructure setup considerations no longer serve as decisive factors in determining SHA acceptance. In current markets, prominent barriers are framed less in terms of one-time installation effort and more in terms of risks, distrust, and limited perception of the overall value of smart homes [10,19,94]. In parallel, users tend to treat initial setup as a hurdle that can be overcome with vendor support or packaged solutions [7,42]. MM likewise exhibited no significant path to PU or IU. This suggests that ongoing management and maintenance tasks, while sometimes inconvenient, do not strongly differentiate SHA acceptance. In this study, MM mainly captures routine upkeep activities. Such activities are often gradually incorporated into everyday practice and treated as part of the normal background work of keeping digital devices running, rather than as a central criterion for evaluating the overall usefulness of SHA [7,42]. As long as core functions continue to work and deliver visible benefits in daily life, households tend to tolerate a certain level of troubleshooting and minor maintenance without fundamentally revising their assessments of the technology [96]. Because users see PS and MM as manageable and part of normal use, they do not rely on them when deciding SHA acceptance.

Unlike the other two dimensions of PDU (PS, MM), AC has a negative effect on both PU and IU. This indicates that when users find it difficult to design or configure automation, they do not simply hesitate to use SHA but begin to doubt whether SHA is genuinely useful. In practice, the configuration phase is where user needs must be translated into concrete trigger-action rules that operate reliably in everyday situations. Many non-expert users find it difficult to understand rule semantics, anticipate exceptional cases, and predict how multiple automations will interact, which often results in misconfigurations, unexpected behaviors, and frustration [59,97–100]. In addition, a large proportion of user support requests concern automation configuration and debugging, and current tools detect only a small subset of configuration issues and rarely suggest concrete fixes, indicating that configuration remains technically and cognitively demanding for ordinary users [50,57]. When users repeatedly face such problems, they are less likely to believe that SHA will reliably save effort or improve their daily routines, which directly undermines their PU. This is consistent with recent technology acceptance research on digital and other high-technology services, which shows that more difficult or effortful interaction tends to lower users’ PU of the technology [101–104]. In other words, AC can be seen as the most critical point of PDU in SHA.

#### Relationship between control variables and intention to use.

ESHA showed a positive effect on IU. Users who have already tried SHA tend to feel more confident about what these technologies can and cannot do, and they form more realistic expectations about performance and everyday fit, which in turn supports a stronger IU [25,105]. Such experience helps users reduce uncertainty, understand the actual benefits and limitations of the system, and judge more clearly whether SHA fits their daily routines. When uncertainty decreases and the expected value becomes clearer, users are naturally more willing to continue using or expand their use of SHA, which explains the higher IU among experienced users. IPSP had positive effects on both PU and IU. Users who are generally interested in products and services for personalization are more likely to view SHA as a useful way to tailor their living environment to individual habits and preferences, and they are also more inclined to accept such services, which align with recent findings on smart and connected services [106–108]. Finally, age and gender did not show significant effects on either PU or IU in this study. Although this should not be assumed to apply universally across all SHA contexts, it aligns with a broader tendency in TAM research where socio-demographic variables often explain little additional variance in IU once core beliefs such as PU are taken into account [105].

### Managerial implications

#### Reframing value propositions through personalization and context awareness.

The non-significant effect of SR on both PU and IU suggests that automation based solely on fixed schedules may no longer meet user expectations in SHA. Users now appear to value more dynamic and responsive interactions rather than simple repetition [109]. Context-aware functionalities, which adjust device behavior based on real-time inputs such as location, time, sensor data, or historical usage, align more closely with current needs [110,111]. For instance, blinds that close automatically when sunlight exceeds a certain level or ventilation systems that activate following cooking activity can help users experience the system as intelligent and adaptive. Emphasizing such responsiveness may shift user expectations away from static automation toward more fluid and responsive services.

In contrast to SR, IPSP demonstrates a significant and positive effect on both PU and IU, indicating that users place higher value on personalization in shaping both their evaluations and acceptance. Personalization allows users to define lighting scenes for particular activities, configure voice commands, and tailor environmental settings based on individual preferences, thereby enhancing both system relevance and perceived autonomy. These findings imply that the core value proposition of SHA should be reoriented toward personalization [108]. SHA providers are encouraged to embed personalization as an essential component of design, ensuring that users perceive SHA not merely as an automated system, but as a flexible and user-responsive service. Promoting those aspects of SHA through marketing can further support acceptance, particularly among users who seek services aligned with their unique routines and lifestyles.

#### Considerations for strengthening perceived usefulness: insights from energy-saving and lack of time for household tasks.

The finding that ES significantly increases PU, while showing a negative effect on IU, indicates that users with ES can recognize the usefulness of SHA but may require additional support to translate this recognition into action. Particularly in regions where electricity costs are relatively low, financial savings alone may not offer sufficient incentive [85,94,112]. Therefore, service providers may consider framing energy-related functions as sources of convenience and environmental contribution. For example, real-time dashboards that visualize consumption trends and notify users of wasteful usage from connected devices may enhance perceived usefulness by combining ecological awareness with practical insights [113–115].

In parallel, the strong influence of LT on IU, despite its lack of association with PU, suggests that users with limited time resources may use SHA out of necessity rather than through a structured understanding of usefulness. For such users, clearly communicating how SHA reduces daily burdens through automation may enhance their PU. Dashboards that quantify time saved, summarize completed routines, or visualize reductions in manual effort could serve this purpose. These strategies collectively emphasize the need to strengthen PU by aligning system design and communication with users’ environmental interests and situational needs, thereby supporting more consistent and scalable acceptance.

#### Designing for ease of use: insights from perceived difficulty of use.

The non-significant effect of PS on PU and IU suggests that simply owning compatible devices does not facilitate perceived value or encourage usage. As ICT-based smart devices have become increasingly accessible, their physical presence in households may be considered an accessible condition [46,116]. However, even though users may already possess the necessary hardware, they may still lack sufficient clarity regarding how devices coordinate with one another, especially in cases requiring multi-device interaction. Such limitations appear closely related to the observed negative influence of AC on both PU and IU, suggesting that users not only have trouble in rule creation but also in comprehending how various components can be orchestrated meaningfully. The cognitive burden associated with defining conditions, selecting appropriate combinations of devices, and managing complex scenarios may negatively affect PU and IU. Therefore, SHA providers should prioritize supportive design features such as preconfigured automation templates, adaptive scenario suggestions based on contextual usage patterns, and step-by-step configuration wizards [57,117–119]. These tools allow users to initiate relevant automation with minimal effort, lowering entry barriers and reducing reliance on technical knowledge. In addition, usage analytics can help identify stages where users drop off, thereby guiding improvements to onboarding and interfaces.

Although MM was not found to significantly influence PU or IU, it remains an important consideration for long-term user satisfaction. While maintenance-related demands may not be prominent during initial evaluation, accumulated issues such as delayed updates, troubleshooting difficulties, or the frustration and fatigue caused by failed automation configurations may over time diminish sustained use. Consequently, service providers are encouraged to implement automated updates, self-diagnosis functions, and transparent system health indicators to reduce post-acceptance burdens.

#### Segmentation and familiarity-based strategies.

The findings of this study suggest that SHA acceptance can be more effectively supported when outreach strategies are tailored to particular user characteristics. Segmentation based on UL and PDU enables service providers to better align SHA with the needs and preferences of distinct user groups [25,26]. For instance, users with high ES may be more responsive to communication that emphasizes environmental contribution or sustainability framing, while users with high LT are more likely to respond to messaging that highlights time savings or reduced household task burden. Users with high IPSP typically expect a high degree of personalization, making flexible customization and user-defined control essential. In practical applications, such users may be identified through behavioral patterns, including frequent interaction with personalization features in mobile applications or digital content platforms. These segmentation-based insights offer a more predictive and actionable foundation for service design and communication planning.

In addition to segmentation-based strategies, the significant positive effect of ESHA on IU highlights the role of prior familiarity in encouraging confident use. Structured onboarding programs that include prebuilt automation scenarios, shared user experiences, and gradually introduced advanced functions may help convert familiarity into active use. Reinforcing familiarity through carefully designed early-stage interactions remains a key consideration for enhancing SHA acceptance [103,109,120].

## Concluding remarks

This study empirically validates the influence of users’ lifestyles (UL) and perceived difficulty of use (PDU) on smart home automation (SHA) acceptance. By integrating these two user-centered constructs into the analysis, the study addresses a gap in prior research, which has predominantly emphasized technological attributes or demographic characteristics while giving limited attention to lifestyle-driven acceptance drivers and difficulty-related barriers. The principal contribution of this research lies in the comprehensive model that systematically examines SHA acceptance through UL and PDU. The research model validates that UL, operationalized through energy-saving, structured routines, and lack of time for household tasks, significantly influences intention to use (IU). Simultaneously, the model demonstrates how PDU, measured across product and infrastructure setup, automation configuration, and management and maintenance, affects acceptance. Accordingly, this research offers a structured framework in which UL and PDU are decomposed into theoretically grounded dimensions and jointly modelled as external drivers of perceived usefulness (PU) and IU for SHA. This structured approach provides researchers with precise instruments to analyze the UL and PDU dimensions affecting SHA acceptance. The research model advances theoretical understanding by demonstrating that UL and PDU distinctly influence PU and IU, thereby clarifying their respective roles in SHA acceptance. This framework serves as a foundation for future studies in related smart service domains.

In addition to its theoretical contributions, the study offers actionable insights for SHA providers. These include reframing value propositions to highlight personalization and context awareness, strengthening PU, reducing PDU through simplified configuration, and applying user-centered segmentation. These directions reflect the importance of aligning service design and communication with users’ expectations. The identification of indirect effects, particularly the mediating role of PU in linking UL and PDU to IU, highlights how users move from recognizing PU to forming IU. These findings provide a basis for more targeted service design and improvement strategies, helping to support not only initial acceptance but also sustained and meaningful use of SHA.

While this study provides valuable findings, several limitations should be acknowledged. First, although the sample size of 167 respondents was sufficient for statistical analysis, the relatively limited sample scope may constrain the generalizability of results. The findings may reflect characteristics of a particular population within a defined context rather than being representative of the broader SHA user base. Future studies should validate the proposed framework using larger and more diverse samples to improve external validity.

Second, this study adopted a first-order factor model for UL and PDU to enable dimension-level analysis aligned with the research question. However, both constructs are inherently multidimensional. Future research may benefit from testing second-order or bifactor structures to capture how different sub-dimensions uniquely influence PU and IU. A more complex modeling approach could offer deeper insights into the internal structure of UL and PDU, especially as SHA becomes more widely accepted.

This study also suggests several promising avenues for future research. First, future studies should examine how UL and PDU manifest differently across housing environments and cultural contexts. For example, in high-density, apartment-based settings such as South Korea, where centralized systems are common, users may experience PDU differently from those in low-density, single-family housing regions like the United States or Australia. Regionally adaptive models that account for infrastructure and sociocultural norms will help improve the explanatory power of SHA acceptance research.

Second, future work should explore SHA’s integration with complementary services such as energy management, eldercare, and security. These cross-domain linkages introduce new sources of perceived value as well as increased complexity. For instance, SHA linked with energy analytics or health monitoring may appeal to new lifestyles but also increase PDU due to multi-system interaction. Capturing these effects may require refining UL and PDU constructs to reflect domain-related user experiences and interaction complexities.

Third, the role of emerging technologies, especially artificial intelligence (AI), warrants deeper investigation. AI-enabled automation can lower perceived effort by learning user preferences and managing configurations dynamically. In parallel, trust in AI-driven systems may moderate how users evaluate usefulness and form IU. Future research should examine how user trust in AI influences PU and IU in SHA, as well as how transparent and reliable AI behaviors affect acceptance and long-term use.

By addressing these limitations and expanding along the suggested research directions, future studies can build on the current framework to develop a more comprehensive understanding of SHA usage. This will support the design of user-centered, sustainable, and scalable solutions that facilitate both initial acceptance and continued use with SHA.

### Declaration of Generative AI and AI-assisted technologies in the writing process

During the preparation of this work the authors used ChatGPT in order to check grammar, spelling, translation and academic tone. After using this tool/service, the authors reviewed and edited the content as needed and take full responsibility for the content of the publication.

## Abbreviations

SHA: Smart home automation
UL: Users’ lifestyles
PDU: Perceived difficulty of use
PU: Perceived usefulness
IU: Intention to use
ESHA: Prior experience with SHA
IPSP: Interests in products and services for personalization
